# Reaching out, inviting back: using Interactive voice response (IVR) technology to recycle relapsed smokers back to Quitline treatment – a randomized controlled trial

**DOI:** 10.1186/1471-2458-12-507

**Published:** 2012-07-06

**Authors:** Beatriz H Carlini, Anna M McDaniel, Michael T Weaver, Ross M Kauffman, Barbara Cerutti, Renée M Stratton, Susan M Zbikowski

**Affiliations:** 1 Alere Wellbeing, Seattle, 999 Third Avenue, Suite 2100, Seattle, WA, 98104-1139, USA; 2 Alcohol and Drug Abuse Institute (ADAI), University of Washington, 1107 NE 45th St, Suite 120, Seattle, WA, 98105, USA; 3 Indiana University School of Nursing, 1111 Middle Drive NU340e, Indianapolis, IN, 46202, USA; 4 Bluffton University, 1 University Drive, Bluffton, OH, 45817-2104, USA

## Abstract

**Background:**

Tobacco dependence is a chronic, relapsing condition that typically requires multiple quit attempts and extended treatment. When offered the opportunity, relapsed smokers are interested in recycling back into treatment for a new, assisted quit attempt. This manuscript presents the results of a randomized controlled trial testing the efficacy of interactive voice response (IVR) in recycling low income smokers who had previously used quitline (QL) support back to QL support for a new quit attempt.

**Methods:**

A sample of 2985 previous QL callers were randomized to either receive IVR screening for current smoking (control group) or IVR screening plus an IVR intervention. The IVR intervention consists of automated questions to identify and address barriers to re-cycling in QL support, followed by an offer to be transferred to the QL and reinitiate treatment. Re-enrollment in QL services for both groups was documented.

**Results:**

The IVR system successfully reached 715 (23.9%) former QL participants. Of those, 27% (194/715) reported to the IVR system that they had quit smoking and were therefore excluded from the study and analysis. The trial’s final sample was composed of 521 current smokers. The re-enrollment rate was 3.3% for the control group and 28.2% for the intervention group (p < .001). Logistic regression results indicated an 11.2 times higher odds for re-enrollment of the intervention group than the control group (p < .001). Results did not vary by gender, race, ethnicity, or level of education, however recycled smokers were older (Mean =45.2; SD = 11.7) than smokers who declined a new treatment cycle (Mean = 41.8; SD = 13.2); (p = 0.013). The main barriers reported for not engaging in a new treatment cycle were low self-efficacy and lack of interest in quitting. After delivering IVR messages targeting these reported barriers, 32% of the smokers reporting low self-efficacy and 4.8% of those reporting lack of interest in quitting re-engaged in a new QL treatment cycle.

**Conclusion:**

Proactive IVR outreach is a promising tool to engage low income, relapsed smokers back into a new cycle of treatment. Integration of IVR intervention for recycling smokers with previous QL treatment has the potential to decrease tobacco-related disparities.

**Trial registration:**

ClinicalTrials.gov Identifier: NCT01260597

## Background

Tobacco dependence is a chronic, relapsing condition that typically requires multiple quit attempts and extended treatment, including behavioral counselling and pharmacotherapy [1].. A number of studies in the literature document relapsed smokers’ interest in recycling back into treatment for a new, assisted quit attempt when offered the opportunity [2-9].

The lack of procedures available to routinely re-engage individuals who relapse might negatively impact low income smokers more than other socioeconomic groups. Relapse rates among low income smokers are high due in part to obstacles related to maintaining abstinence while working and socializing in environments that are conducive to tobacco use [10-14]. Medicaid recipients and uninsured individuals, two prominent low income populations in the US, have higher rates of smoking than privately insured individuals. National data indicate that while 18% of insured adults are current smokers, 37% of Medicaid recipients and 3% of the uninsured smoke [15].

Smokers covered only by Medicaid or with no insurance are much less likely to successfully quit than privately insured smokers [14]. State funded tobacco quitlines (QLs) provide evidence-based, free cessation treatment for Medicaid and uninsured smokers [16], but they typically do not proactively reach out to their former participants to encourage those who relapsed to re-enroll in services.

Proactive interventions promoting re-engagement into a new cycle of treatment for low income QL participants who did not quit or are relapsed have potential to offset tobacco-related disparities.

Carlini et al. [9] found that 44.7% of low income smokers from ethnic populations who previously used QL services opted to re-engage into a new cycle of QL support when proactively reached and invited to participate by QL staff. Meanwhile, only 0.5% of the control arm of the study spontaneously re-enrolled in QL support for a new quit attempt in any given month [9]. This intervention, although effective, would be too expensive for large scale dissemination.

Interactive voice response (IVR) systems are capable of proactively contacting individuals by telephone and communication technologies exist to provide messages tailored to an individual needs, which may provide an efficient and effective means of motivating low income smokers to re-enroll in QLs.

This manuscript describes the results of a randomized controlled trial testing the efficacy of IVR interventions to recycle smokers who used a QL in the past back to QL support for a new quit attempt. It was hypothesized that the rates of recycling into treatment among those receiving the IVR intervention would be higher than smokers who were not proactively invited to re-engage in QL services.

## Methods

### Development of the IVR intervention

IVR technology is a feasible and cost-effective method for conducting health-related research (e.g., clinical trials, evaluation) and aiding patient care (e.g., disease management, psychological assessment, monitoring substance use, and medication adherence). IVR systems can be programmed to ask a series of pre-recorded questions to collect data from individuals via a touchtone telephone keypad. A computer algorithm controls the sequencing of questions and provides scripted feedback based on the user’s input. IVR systems can also be programmed to make proactive calls at predetermined times and intervals to a large population of individuals without the use of time and resource intensive staff (e.g., counselors, nurses, and research assistants). The IVR intervention utilized in this trial was developed in two steps. The first step focused on creating the content of the IVR messages. Four prototype IVR messages about possible barriers to re-engagement in QL support for quitting smoking were developed, based on previous work with low income ethnic/racial minority smokers [9]. These prototype messages were tested and changed according to feedback received through individual telephone interviews with fifteen Medicaid insured and uninsured smokers who had previously used a QL and agreed to be contacted further. The messages aimed to a) redefine relapse as a learning opportunity and not as a failure; b) motivate new quit attempts by reminding smokers about benefits in quitting (e.g., personal health and wellbeing, financial savings, concern for family members); c) educate smokers about the different offerings of QL support services; d) reiterate how QL support can increase the chances of quitting; and e) inform smokers of their eligibility to re-enroll in QL services. The full content of the barriers assessment and tailored messages are given in Additional file 1.

The second step was a usability test of a prototype IVR system. Fifteen additional low income smokers were recruited from a pool of recent QL services users to test the IVR system prototype by responding to questions by pressing options chosen by the investigators. This strategy assured that every combination of the IVR system options was tested by at least two smokers. The usability test was followed by a 15-minute telephone interview with one of two investigators (BHC and RMK). A semi-structured interview script was used to collect feedback on message length, content clarity, voice, tone of the IVR message, and usability of the system. Results of the usability testing informed the modifications made to the IVR intervention that was ultimately used in this trial. The call diagram is presented in Additional file 2.

### Design

Two-arm, randomized, controlled trial.

### Study population

The target population of the study was low income smokers, defined as being a Medicaid recipient or uninsured by the time of their initial enrollment into quitline treatment. Medicaid is a program designed to assist individuals and families with low incomes and limited resources. In 2009, 20% of the US population received some kind of Medicaid benefit (http://www.statehealthfacts.org/index.jsp). Among the groups of people served by Medicaid are eligible low-income parents, children, seniors, and people with disabilities. Medicaid is the largest source of funding for medical and health-related services for people with limited income. In 2009, 16% of the US population was uninsured (http://www.statehealthfacts.org/index.jsp); 40% of uninsured have incomes less than 100% of the Federal Poverty Level (FPL) as measured by the U.S. Department of Health and Human Services' (HHS) poverty guidelines.

### Sample

The sample was comprised of all participants who enrolled in Indiana (IN) or Washington (WA) QLs from June to September of 2009 and were: Medicaid or uninsured, age 18 or older; received services in English; provided consent to be contacted by the QL for follow-up; and sought help primarily for cigarette use (use of other forms of tobacco had to be secondary to the use of cigarettes). In Indiana, 10.9% of the population received Medicaid benefits and 11.6% were uninsured. In Washington, these percentages are 12.6% and 11.7% respectively (http://www.statehealthfacts.org/index.jsp, data for 2009).

### Recruitment

**Figure 1:**
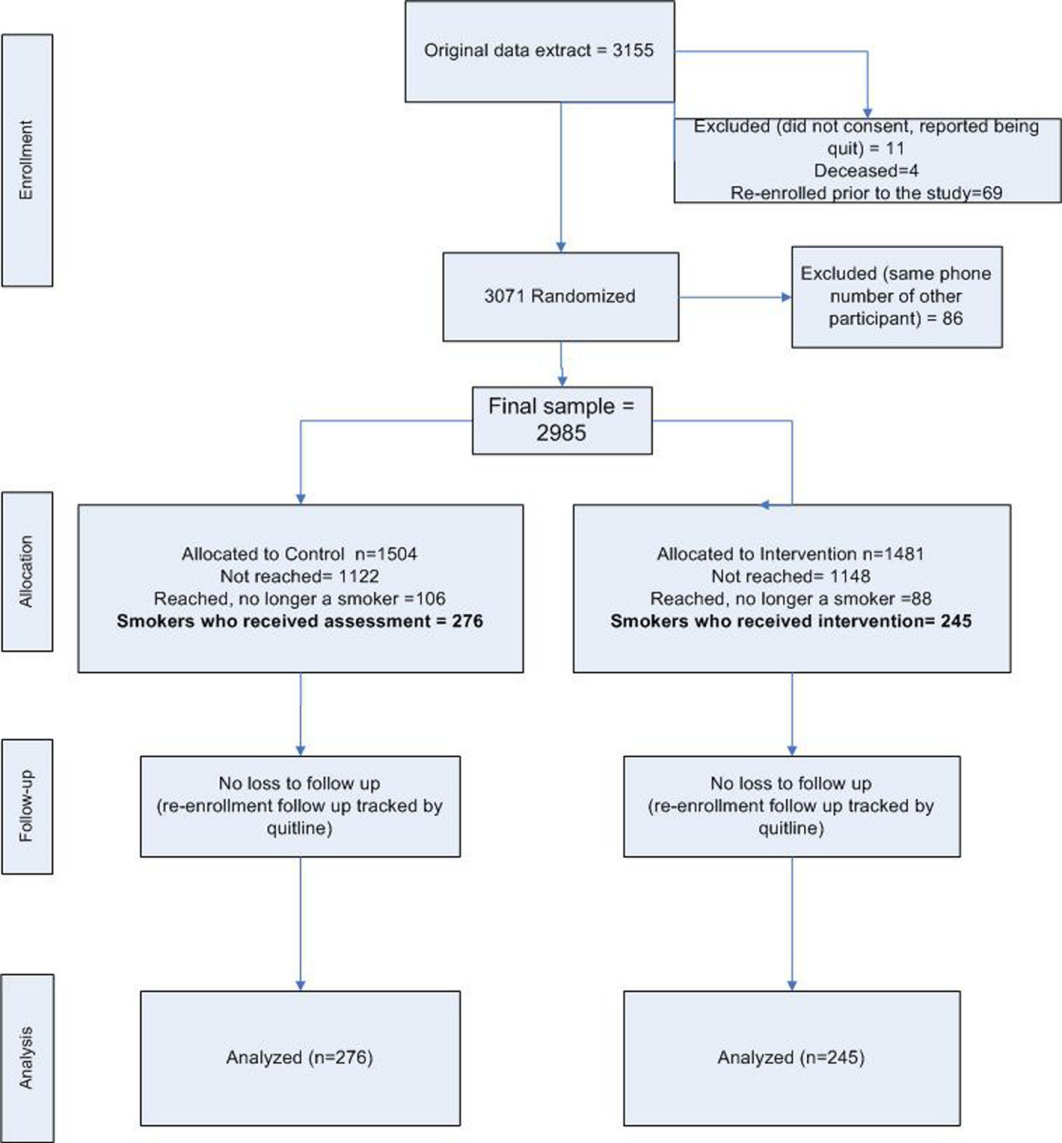
CONSORT diagram.

Letters describing the study and instructions of how to opt out were sent to the 3155 individuals who met the eligibility criteria one month prior to the launch of the study. Those participants who did not proactively call, email, or send an opt-out letter to the research team requesting to be dropped from the study were included in the study. The final number of individuals called by Silverlink, the IVR vendor, was 2985 (Figure 1).

The study procedures were approved by Indiana University and Western Institutional Review Boards.

### Study Arms

#### Intervention condition

The IVR intervention had four components. The first component provided a salutation and authentication, explanation of the purpose of the call, and elicited data to identify the recipient as the targeted, former QL participant. Next, the system screened for current tobacco use. Respondents who indicated being abstinent (i.e., 30-day point prevalence abstinence and therefore not eligible for the intervention) received a brief congratulatory message and were excluded from the trial. Respondents who indicated current smoking continued to a third component of the IVR intervention and were asked to identify barriers to their re-engagement in QL support and the IVR system then provided brief tailored messages to specifically address those barriers. The final component of the IVR call, offered at the completion of each set of questions and at the end of the intervention, was an automated transfer to a “live” QL registration specialist. Those who were interested in re-enrolling into QL treatment but were not willing or able to do it at the time of the IVR call were able to leave their contact information and schedule a call back by the QL. Those who reported being quit were excluded from analysis.

#### Control condition

Participants randomized to the control condition received only the first two components of the IVR intervention (greeting and screening of smoking status), followed by a message thanking them for the information. Those who reported being quit were excluded from analysis.

### Procedures

Eligible participants were randomized to the intervention or usual care prior to entry into the IVR calling database. The IVR system attempted to reach participants in both groups up to twenty times. The attempts were delivered in two phases and delivered over non-consecutive days: ten attempts over the course of four weeks to participants’ primary phone number, followed by another round of ten attempts to participants’ secondary phone number (if secondary number was not provided, the extra attempts were made to the primary number). An error prevented 699 participants from receiving that second round of ten attempts. These participants were less likely to be reached than those receiving the full course of twenty attempts (29.2% vs 38.2%, p < 0.005).

A call was considered completed and no longer attempted if the participant confirmed to the IVR system that he/she was the intended recipient of the call (‘authentication’). All attempts and IVR assessment and interventions were delivered by Silverlink between September 22 and November 5, 2010.

### Measures

#### Demographic characteristics and tobacco history

Gender, age, race, ethnicity, insurance type, education, chronic conditions (Diabetes, Asthma, Chronic Obstructive Pulmonary Disease –COPD, and Coronary Artery Disease- CAD), lifetime and current smoking, cigarettes smoked per day, history of quit attempts, previous use of tobacco cessation treatments, and use of other tobacco products were obtained from participants’ previous QL registration records.

#### Participants reached

The IVR system provided reports on participants reached. Reach was defined as having a call “authenticated” (confirmation that the intended recipient had answered the call) and a valid response to the first IVR question, on current smoking status.

#### Smoking status

The IVR system collected updated information on participants’ quit status. Those who were reached and reported being quit were excluded from the study.

Two other sets of measures were obtained from smokers who were reached by the IVR system.

#### *Barriers to re-*enrollment *in QL*

The IVR system collected and then provided reports on participants’ reported barriers to re-enrollment in treatment. The wording and logic of the barrier items utilized in the study can be seen in Additional file 1 and Additional file 2.

#### Re-enrollment into QL support

This was the main outcome of the study and was obtained by consulting QL registration files and IVR reports. Re-enrollment was defined as new registration in QL support from study launch (September 22, 2010) to one month after the last attempt of reaching participants (December 4^th^, 2010).

### Analysis

All analyses were conducted using SAS version 9.2 [17]. Descriptive statistics, including means, standard deviations, ranges, frequencies, and percents were produced and examined for value distribution, out-of-range, and patterns of missing. Differences in demographic variables between treatment groups were examined using a contingency table analysis and *χ*^2^ (nominal variables) or t-tests (continuous variables) to identify potential covariates for use in logistic regression models comparing treatment groups. Due to relatively small cell sizes, race/ethnicity was dichotomized into two groups, namely Non-Hispanic White and Other, for inferential analyses. A propensity score, representing predicted probability for being reached, was derived using an all subsets logistic regression approach. The propensity score was used as a covariate in logistic regression models comparing treatment groups in order to control for potential differences in reaching participants between the two groups. Bivariate associations between re-enrollment and selected nominal variables were tested using a contingency table analysis and *χ*^2^. Treatment effects on re-enrollment were also tested using a logistic regression model in order to control for selected demographic differences between treatment groups. Covariate interactions with treatment were tested in the logistic regression models using change in −2 log likelihood, and removed if not statistically significant. Overdispersion was tested in the logistic regression models, and either Pearson or Deviance scaling applied, as appropriate [18]. Statistical assumptions were tested for each model, and appropriate transformation (e.g., Box-Cox family transform) or robust statistics (e.g., exact p values) employed as needed [19]. Goodness of fit for logistic regression models was tested using the Hosmer-Lemeshow Goodness-of-Fit test [18]. All inferential tests were evaluated using a .05 level of significance.

## Results

The IVR system successfully reached 715 (23.9%) participants. Of those, 194 individuals reported to the IVR system that they had quit smoking (defined by “not a single puff in the last 30 days”) and were therefore excluded from the study. The trial final sample was comprised of 521 current smokers.

The main reasons of not reaching participants were: calls not answered (57%) and hang ups right after authentication (12.2%). Disconnected numbers represented a relative small percentage (6.8%).

### Sample characteristics: original sample and smoker’s trial sample

Table 1 presents demographic and smoking characteristics of the original sample and the trial final sample (participants who were reached by IVR intervention and reported being smokers). The original sample and the smokers participating in the study were similar in terms of several characteristics: about 60% women, mostly white non-Hispanics, with relative low education, and the vast majority daily smokers who reported having their first cigarette within 30 minutes of waking.

**Table 1:** Demographic and smoking characteristics of the original sample and trial final sample (smokers only)

	**Former QL participants randomized (%)**			**Smokers reached and included in the study (%)**		
	**N = 2985**			**n = 521**		
	**Control n = 1504**	**Intervention n = 1481**	**P value**	**Control n = 276**	**Intervention n = 245**	**P value**
**Gender**			.329			.314
Male	40	38.2		38	33.5	
Female	60	61.8		62	66.5	
**Age**						
Mean age (years)	39.9 (13)	39.1 (12.7)	**.101**	42.9 (13.2)	42.2(12.6)	**.543**
**Race/ethnicity**			.350			.970
White, non Hispanic	79.5	78.3		81.2	82	
African American	8.1	8.5		5.8	5.7	
Latino/Hispanic	4	3.4		4.4	3.3	
Native Am./Pacific Isl.	3.5	4		2.9	3.7	
Asian	0.8	1		0.7	1.2	
Other	4.1	4.7		5.1	4.1	
**State**			.501			1.00
Washington	61	59.8		62.7	62.8	
Indiana	39	40.2		37.3	37.1	
**Insurance**			.534			.216
Medicaid	51.6	50.4		53.6	59.2	
No insurance	48.4	49.6		46.4	40.8	
**Education**			.478			.800
Less than HS	25.6	25.4		24.6	29.5	
GED	10.3	10.2		11.7	9.1	
HS degree^b^	54.2	56.2		55.3	53.5	
College degree	9.9	8.2		8.4	7.9	
**Cigarette use**						
Daily	94.9	94.6	.742	93.8	95.0	.572
Mean cigs per day (SD)	20.5 (11.8)	19.6 (12)	.052	**21.5 (12.3)**	**18.8 (12.3)**	**.015**
1st cigarette within 30 min of waking	83.7	83.5	.880	86.7	81.0	.087
**Chronic conditions**			.452			**.022**
one or more	37.7	39.1		**42.4**	**52.6**	
None	62.3	60.9		57.6	47.4	

There were no statistical differences between intervention and control groups for the variables measured in the study and original sample (Table1). The control and intervention arms of the final sample (smokers successfully reached) were statistically different in two domains. The control group had higher mean number of cigarettes smoked per day) than the intervention group and lower prevalence of chronic conditions than the intervention group (Table 1).

### Outcome- re-enrollment in QL support

The re-enrollment rate was 3.3% (9/276) for the control group participants and 28.2% (69/245) for the IVR intervention group (*χ*^2^ = 63.23, DF = 1, p < .001). Out of the 69 re-enrollees in the intervention group, 66.7% (n = 46) accepted a transfer to the QL and registered in services immediately after the IVR call. The remaining 23 participants provided contact information and registered in services when QL staff returned their voice mail. Age, gender, education, health insurance and cigarettes per day were not associated with higher re-enrollment rates after receiving IVR intervention. (*χ*^2^ = 5.06; DF = 4; p = .281). However, participants who accepted re-enrolment were older than those declining a new treatment cycle (Mean = 45.2 years of age; SD = 11.7 vs. 41.8; SD = 13.2; p = 0.013) and more likely to report a chronic condition (60.9% versus 43.4%; p = .001).

Logistic regression results testing the IVR intervention effect on re-enrollment are presented in Table 2, utilizing the final model analysis described in the Methods section. IVR group participants had an 11.2 times higher odds for re-enrollment than the control group (p < .001), after controlling for chronic condition, cigarettes per day, age, and propensity of being reached score (see analysis).

**Table 2:** Logistic regression model testing IVR effect on QL treatment re-enrollment

**Effect**	**b (SEb)**	**OR (95% CI)**	**Chi Squared (DF)**	**p**
Intercept	−0.912 (.649)	N/A	1.97 (1)	.160
Study Arm (Intervention)	2.42 (.372)	11.2 (5.4 - 23.3)	42.4 (1)	<.001
One or more chronic condition	0.720 (.298)	2.0 (1.1 - 3.7)	5.8 (1)	.016
Age	0.036 (.017)	1.04 (1.0 - 1.1)	4.2 (1)	.040
Propensity Score	−5.17 (2.54)	0.006 (<0.01 - 0.84)	4.1 (1)	.042

Participants reporting a chronic condition had a 2.0 times higher odds of re-enrolling in QL support than those who did not report a chronic condition (p = .016), independent of intervention group, age, and propensity score. To explore associations between individual chronic conditions and odds for re-enrolling, presence of selected individual chronic conditions (asthma, coronary artery disease, COPD, and diabetes) were placed in the logistic regression model. Of the set of chronic conditions, only asthma was independently associated with re-enrollment after controlling for all other variables in the model (p = .039; OR = 1.81; 95% CI = {1.03 – 3.2}).

### Reported barriers for re-enrollment

Table 3 describes the re-enrollment barriers captured by the IVR system and endorsed by the intervention group and its subsequent re-enrollment rates.

**Table 3:** Smokers reported barriers to re-enroll in QL support (n = 245)

**Barriers**	**Agree with statement %(N)**	**Re-enrolled %(N)**
*Related to interest in quitting*^a^		
"I am ready to quit"	33.5% (71)	56.3% (40) *
"I would like to quit but tried in the past and couldn’t do it"	36.8% (78)	32.0% (25)
"I am not interested in quitting now"	29.7% (63)	4.8% ** (3)
*Related to QL services*		
"I used the QL support in the past; I am not sure I am eligible to use the QL again"	9% (22)	54.5%* (12)
"I already used the QL; I want something different"	6.5% (16)	43.8% (7)
"I don't see how the QL can help me quit"	5.7% (14)	64.3% * (9)

(a) 33 participants did not respond to this question.

(*) more likely to re-enroll in services than those who did not agree with statement p < 0.05.

(**) less likely to re-enroll in services than those who did agree with statement p < 0.05.

Most respondents reported they “would like to quit” but felt they “couldn’t do it.” After receiving the automated message normalizing relapses and encouraging another attempt (Additional file 1), 32.1% re-enrolled in services. A second frequently reported barrier was “not being interested in quitting”. It is noteworthy that 4.8% of those re-enrolled in services after the delivery of a brief automated message about the benefits of quitting (Additional file 1) and an offer to re-enroll in QL support.

Few smokers (5.7 to 9%) reported agreement with barriers related to QL services *per se*, such as eligibility, uncertainty of how QL could help, or desire for different kinds of support. Those who endorsed the first two barriers and had the chance to listen to the brief IVR message were more likely to re-enroll in QL services than those who did not endorse these statements.

About a third of participants (33.5%) reported being “ready to quit.” In these cases, they were congratulated and offered re-enrollment immediately. Over half re-enrolled in QL support (56.3%).

## Discussion

This study is consistent with previous research in which smokers who utilized cessation support in the past, when proactively reached and offered an opportunity to recycle in treatment, display high rates of treatment re-engagement [2-9].

The major hypothesis of the current study was that proactive automated calls utilizing IVR technology would enhance recycling in treatment among Medicaid and uninsured former QL participants. This hypothesis received strong support as smokers reached by the IVR intervention were 11.2 times more likely to re-enroll in a new treatment cycle than the control group. This was an unexpectedly large odds ratio for the intervention effect, and the 95% confidence interval was also relatively large (5.4-23.3). The overall proportion of re-enrollment was 15% (78/521), while the unadjusted proportion of treatment arm smokers who re-enrolled was 28.2% (69/245), compared to the 3.3% (9/276) of control group smokers re-enrolling. As there was no evidence of limited variability among re-enrolling smokers on the logistic model independent variables (study arm, one or more chronic conditions, age, and propensity score), the most likely cause of the wide confidence interval is the relatively small cell size (N = 9) for control group re-enrollees, which contributes uncertainty to OR estimation. Even with the relatively large confidence interval, the IVR intervention developed here – consisting of assessing barriers for recycling, delivering customized messages to address these barriers, and offering automatic transfer to the QL- was successful in recycling low income, adult smokers irrespective of age, gender, education, and race/ethnic background.

QLs often constitute the only source of professional support available for low income smokers, a segment of society that smokes at higher rates [15] and reports lower success when attempting to quit than middle and high income smokers [14,20]. Integration of IVR technology for re-enrollment procedures/interventions with QL cessation services can address a missed opportunity of reaching out to low income smokers who are satisfied with QL services and want to quit, but do not take the initiative to seek a new assisted quit attempt. Moreover, this can be done in a scalable way, considering that low income QL participants are difficult to reach (i.e., will not answer the telephone, have disconnected numbers on file, or hang up right after answering the call). IVR technology is a viable option to recycle smokers while minimizing the burden to QL staff of making numerous manual calls, thus allowing QL staff to dedicate their time to their primary task of providing smoking cessation counselling. Re-enrollment rates varied according to interest and confidence in quitting. While more than half (56.3%) of smokers reporting being ready to quit registered in QL services, 32% of those interested in quitting but reporting low confidence in their ability to succeed accepted a new cycle of treatment. It is possible that the delivery of a message normalizing relapse and encouraging a new quit attempt helped to achieve this relative high rate of treatment recycling in this group of smokers (see Additional file 1 for IVR message content). Three smokers (4.8%) who reported no interest in quitting chose to recycle back to QL support. This unexpected outcome could be related to a rapid change in interest in quitting, similar to what has been described as “unplanned quit attempts” reported in some population-based studies [21,22]. Alternate explanations for these re-enrollments are that these callers pressed the wrong answer in the IVR system or were reluctant to report interest in quitting before learning more about what would be offered (in terms of pricing, services, or commitment).

Few smokers endorsed barriers to recycle related to QL services (eligibility, effectiveness or relevance). This finding was not surprising considering the study sample consisted of former QL participants, a service that is typically associated with high levels of satisfaction among its users [16].

An important limitation of this study was our inability to reach 76% of participants from the original study sample, somewhat restricting the external validity of our findings. This reach rate is not uncommon in research with low income populations, who tend to switch and disconnect telephone services frequently. However, in this instance, the inability to reach participants was due mostly to calls never answered and not to disconnected numbers. We could have reached more people if our protocol implementation included the entire sample. Unfortunately, a programming error limited our reach by delivering 10 attempts to the primary phone to 699 participants, while the rest of the 2296 subjects in our sample received the full protocol (twenty attempts to the primary and secondary telephone numbers). Another factor that could boost participant reach in the future is the inclusion of caller identification, which would have assisted participants in making a decision on whether or not to take the call. In our study, we utilized a toll-free number not associated with a caller ID. In retrospect, we could have utilized a caller ID that identified the study, potentially increasing the chance of interested smokers accepting the call.

A second limitation relates to the timing of the intervention. Our sample was comprised of smokers who registered in QL support at least 12 months before the study was launched. This choice was made to meet the minimal period required by several states between first and new engagement into QL services. However, some studies suggest that proactive recycling recruitment should be attempted earlier. Fu and colleagues [3] assessed interest among smokers who were treated for tobacco dependence in five Veterans Affairs medical centers. Almost two-thirds of relapsed smokers were interested in recycling into treatment within 30 days. Joseph and colleagues [5] studied interest in further treatment among 2,340 smokers from the Minneapolis Veterans Administration Medical Center who received prescriptions for a smoking cessation aid during an 18-month period. Of continuing smokers, 98% were willing to make another quit attempt—50% immediately, and 28% within 1 month. In a population based recent study, Yeomans et al. [23] found high variability on time elapsed since a new quit attempt among 427 current and former smokers, varying from 1–1162 days. By 6 months, the cumulative proportion of subjects making their next quit attempt was 24.5%, which increased to 52.9% by 12 months (no information is given on what the proportion of these new quit attempts were supported by treatment.). Future studies may want to compare different timing of proactive recycling recruitment of relapsed smokers.

## Conclusion

Proactive IVR outreach is a promising technology to engage low income relapsed smokers in a new cycle of treatment. Integration of QL treatment with an IVR intervention for recycling smokers has the potential to decrease tobacco-related disparities and should be considered as an option by state QLs as a way to increase support for disparate populations such as low income smokers.

## Competing interests

The authors declare that they have no competing interests.

## Authors’ contributions

BHC conceived the study, obtained research funding, supervised the intervention development, developed randomized trial protocol and wrote the manuscript draft. AMM participated in the grant proposal development; AMM and SMZ participated in the conception and study design. AMM, SMZ, RMK, BC and RMS offered feedback during intervention development and assisted on protocol development. RMK developed interview scripts and performed feasibility interviews with study participants. MTW developed the statistical analysis plan and performed the data analysis. BC coordinated every phase of the study, managed the contract with IVR provider, and prepared data for analysis, including quality assurance and data consolidation. RMS managed study-related activities at Indiana University. All authors read and approved the final manuscript.

## Pre-publication history

The pre-publication history for this paper can be accessed here:

http://www.biomedcentral.com/1471-2458/12/507/prepub

## Supplementary Material

Additional file 1 IVR intervention diagram. Click here for file

Additional file 2 IVR-delivered messages to encourage recycling to QL treatment. Click here for file

## References

[B1] Fiore MC Jaen CR Baker TB Bailey WC Benowitz NL Curry SJ Treating tobacco use and dependence: 2008 Update. Clinical practice guideline 2008 US Department of Health and Human Services. Public Health Service, Rockville, MD

[B2] Lando HA Pirie PL Roski J McGovern PG Schmid LA Promoting abstinence among relapsed chronic smokers: the effect of telephone support American Journal of Public Health 1996 86 1786 1790 10.2105/AJPH.86.12.1786 9003138 PMC1380734

[B3] Fu SS Partin MR Snyder A An LC Nelson DB Clothier B Promoting repeat tobacco dependence treatment: are relapsed smokers interested? American Journal of Managed Care 2006 12 235 243 16610925

[B4] Gourlay SG Forbes A Marriner T Pethica D McNeil JJ Double blind trial of repeated treatment with transdermal nicotine for relapsed smokers British Medical Journal 1995 311 363 366 10.1136/bmj.311.7001.363 7640544 PMC2550432

[B5] Joseph AM Rice K An LC Mohiuddin A Lando H Recent quitters' interest in recycling and harm reduction Nicotine & Tobacco Research 2004 6 1075 1077 10.1080/14622200412331324893 15801581

[B6] Partin MR An LC Nelson DB Nugent S Snyder A Fu SS Randomized Trial of an Intervention to Facilitate Recycling for Relapsed Smokers American Journal of Preventive Medicine 2006 31 293 299 10.1016/j.amepre.2006.06.021 16979453

[B7] Tonnesen P Mikkelsen KL Norregaard J Jorgensen S Recycling of hard-core smokers with nicotine nasal spray Eur Respir J 1996 9 1619 1623 10.1183/09031936.96.09081619 8866582

[B8] Tonnesen P Norregaard J Urbain S Simonsen K Recycling with nicotine patches in smoking cessation Addiction 1993 88 533 539 10.1111/j.1360-0443.1993.tb02060.x 8485431

[B9] Carlini BH Zbikowski SM Javitz HS Deprey TM Cummins SE Telephone-based tobacco-cessation treatment: re-enrollment among diverse groups American Journal of Preventive Medicine 2008 35 73 76 10.1016/j.amepre.2008.03.025 18541180 PMC2682706

[B10] Paul CL Ross S Bryant J Hill W Bonevski B Keevy N The social context of smoking: A qualitative study comparing smokers of high versus low socioeconomic position BMC Public Health 2010 10 211 10.1186/1471-2458-10-211 20420707 PMC2868819

[B11] Pisinger C Aadahl M Toft U Jorgensen T Motives to quit smoking and reasons to relapse differ by socioeconomic status Prev Med 2011 52 48 52 10.1016/j.ypmed.2010.10.007 21047525

[B12] Redonnet B Chollet A Fombonne E Bowes L Melchior M Tobacco, alcohol, cannabis and other illegal drug use among young adults: The socioeconomic context Drug Alcohol Depend 2012 121 3 231 239 10.1016/j.drugalcdep.2011.09.002 21955362

[B13] Zhang X Martinez-Donate AP Kuo D Jones NR Palmersheim KA Trends in home smoking bans in the USA, 1995–2007: prevalence, discrepancies and disparities Tob Control 2012 21 3 330 336 10.1136/tc.2011.043802 21813487 PMC5633927

[B14] Lillard DR Plassmann V Kenkel D Mathios A Who kicks the habit and how they do it: Socioeconomic differences across methods of quitting smoking in the USA Social Science & Medicine 2007 64 2504 2519 10.1016/j.socscimed.2007.02.036 17418470 PMC3621978

[B15] Pleis JR Lucas JW Ward BW Summary health statistics for U.S. adults: National Health Interview Survey, 2008. National Center for Health Statistics Vital Health Stat 2009 10 242 20821903

[B16] Maher JE Rohde K Dent CW Stark MJ Pizacani B Boysun MJ Is a statewide tobacco quitline an appropriate service for specific populations? Tobacco Control 2007 16 i65 i70 10.1136/tc.2006.019786 18048635 PMC2598524

[B17] SAS/STAT 9.2 Users Guide 2008 SAS Institute Inc, Cary, NC

[B18] Hosmer D Lemeshow S Applied logistic regression 1989 Wiley & Sons, New York

[B19] Kutner M Nachtsheim C Neter J Li W Applied Linear Statistical Models 2005 5 McGraw-Hill Irwin, Boston

[B20] Hiscock R Judge K Bauld L Social inequalities in quitting smoking: what factors mediate the relationship between socioeconomic position and smoking cessation? J Public Health (Oxf) 2011 33 39 47 10.1093/pubmed/fdq097 21178184

[B21] Ferguson SG Shiffman S Gitchell JG Sembower MA West R Unplanned quit attempts--results from a U.S. sample of smokers and ex-smokers Nicotine Tob Res 2009 11 827 832 10.1093/ntr/ntp072 19509277

[B22] Larabie LC To what extent do smokers plan quit attempts? Tob Control 2005 14 425 428 10.1136/tc.2005.013615 16319368 PMC1748114

[B23] Yeomans K Payne KA Marton JP Merikle EP Proskorovsky I Zou KH Smoking, smoking cessation and smoking relapse patterns: a web-based survey of current and former smokers in the US Int J Clin Pract 2011 65 1043 1054 10.1111/j.1742-1241.2011.02758.x 21923845

