# Supplementary material for: Reaching out, inviting back: using Interactive voice response (IVR) technology to recycle relapsed smokers back to Quitline treatment – a randomized controlled trial
Source: BMC Public Health. 2012 Jul 6;12:507. doi: 10.1186/1471-2458-12-507 (PMC3438078; doi:10.1186/1471-2458-12-507)
Supplement: Additional file 1 — IVR intervention diagram. [file 1471-2458-12-507-S1.doc]

**Appendix 1**

**I would like to quit but tried in the past and couldn’t do it.**

Even if you tried before and didn’t quit for good, you can still quit now. If you are like most people, it may take you a few tries before you quit smoking. The coaches from the QL are ready to help smokers who tried to quit before and want to try to quit again.

**I am not interested in quitting now.**

We know quitting is hard…still, quitting is one of the best things you can do for your health and your family. We are happy to help you with our free services. Our Quit Coaches are experts in helping people quit smoking and are here for you every step of the way. When you are ready to try to quit again, call us at 1-800-QUIT-NOW seven days a week.

**I already used QL support in the past. I am not sure I am eligible to use it again.**

It sounds like you’re wondering if you qualify to use the free services of QL again. You ***can*** use free Quitline services, right now.

**I already used the QL. I want something different.**

It seems like you don’t want the same thing all over again. Maybe you want new medicines to help you quit, or internet support, or more involvement from your friends or family. Well, we have good news: if you want something different from the last time you called the QuitLine, just tell your coach. We will work with you to give you the support you need.

**I don’t see how the QL can help me quit.**

It seems like you don’t see how quitlines can help. Did you know that using the QuitLine services can double your chances of quitting for good? We offer personalized professional support every step of the way. The program is free and you may qualify to have free medicines to help you quit delivered to you.We hope you want to try to quit again using the QuitLine services.
