# Supplementary figures and images for: Reaching out, inviting back: using Interactive voice response (IVR) technology to recycle relapsed smokers back to Quitline treatment – a randomized controlled trial
Source: BMC Public Health. 2012 Jul 6;12:507. doi: 10.1186/1471-2458-12-507 (PMC3438078; doi:10.1186/1471-2458-12-507)

**Appendix 2 – IVR Intervention**


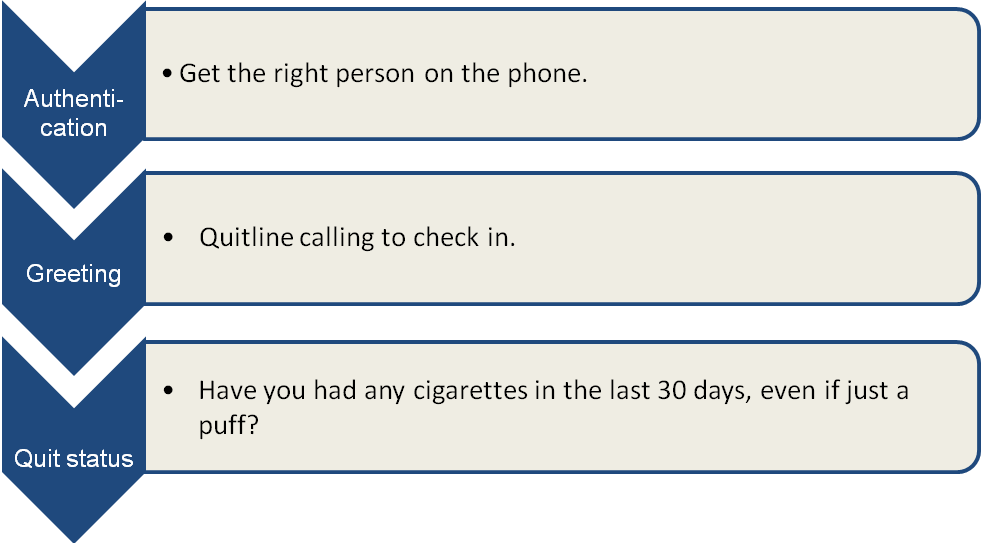


IF SMOKER


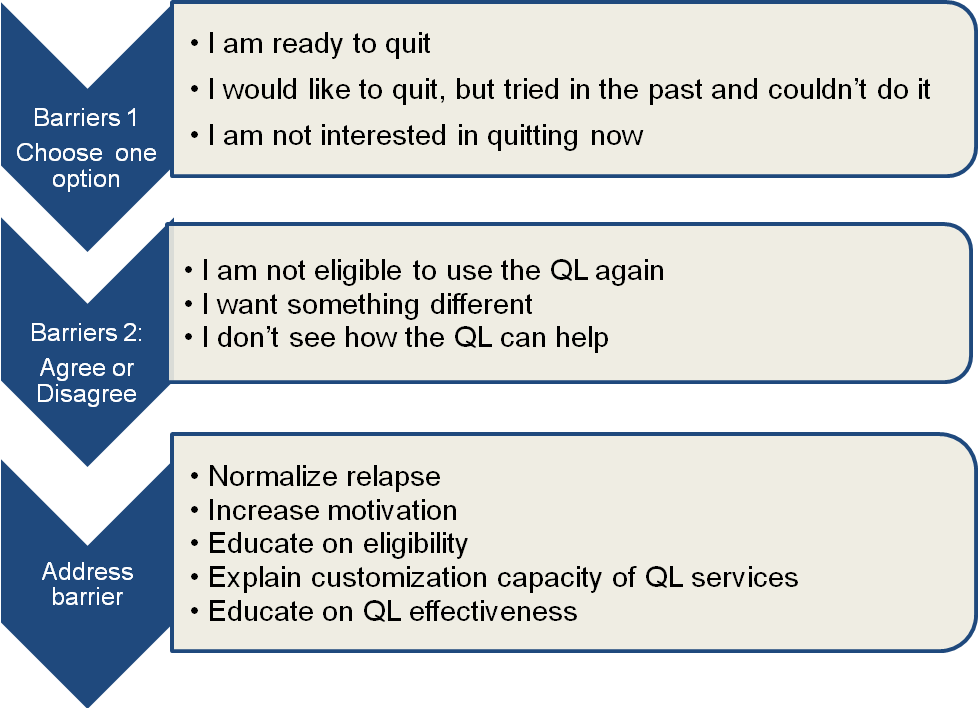


Offer re-enrolment to QL

Supplement: Additional file 2 — IVR-delivered messages to encourage recycling to QL treatment. [file 1471-2458-12-507-S2.doc]
